# Proof of Concept of a Novel Multiepitope Recombinant Protein for the Serodiagnosis of Patients with Chagas Disease

**DOI:** 10.3390/pathogens12020312

**Published:** 2023-02-14

**Authors:** Juliana Martins Machado, Isabela Amorim Gonçalves Pereira, Ana Clara Gontijo Maia, Mariana Ferraz Chaves Francisco, Lais Moreira Nogueira, Isadora Braga Gandra, Anna Julia Ribeiro, Kamila Alves Silva, Carlos Ananias Aparecido Resende, Jonatas Oliveira da Silva, Michelli dos Santos, Ana Alice Maia Gonçalves, Grasiele de Sousa Vieira Tavares, Miguel Angel Chávez-Fumagalli, Mariana Campos-da-Paz, Rodolfo Cordeiro Giunchetti, Manoel Otávio da Costa Rocha, Ana Thereza Chaves, Eduardo Antônio Ferraz Coelho, Alexsandro Sobreira Galdino

**Affiliations:** 1 Laboratório de Biotecnologia de Microrganismos, Universidade Federal de São João Del-Rei, Divinópolis 35501-296, MG, Brazil; 2 Programa de Pós-Graduação em Ciências da Saúde: Infectologia e Medicina Tropical, Faculdade de Medicina, Universidade Federal de Minas Gerais, Belo Horizonte 30130-100, MG, Brazil; 3 Computational Biology and Chemistry Research Group, Vicerrectorado de Investigación, Universidad Católica de Santa María, Arequipa 04000, Peru; 4 Laboratório de NanoBiotecnologia & Bioativos, Universidade Federal de São João Del-Rei, Sebastião Gonçalves Coelho, 400, Divinópolis 355901-296, MG, Brazil; 5 Laboratório de Biologia das Interações Celulares, Departamento de Morfologia, Instituto de Ciências Biológicas, Universidade Federal de Minas Gerais, Belo Horizonte 31270-901, MG, Brazil

**Keywords:** multiepitope, recombinant protein, immunodiagnosis, Chagas disease

## Abstract

Chagas disease remains a neglected disease that is considered to be a public health problem. The early diagnosis of cases is important to improve the prognosis of infected patients and prevent transmission. Serological tests are the method of choice for diagnosis. However, two serological tests are currently recommended to confirm positive cases. In this sense, more sensitive and specific serological tests need to be developed to overcome these current diagnosis problems. This study aimed to develop a new recombinant multiepitope protein for the diagnosis of Chagas disease, hereafter named rTC. The rTC was constructed based on amino acid sequences from different combinations of *Trypanosoma cruzi* antigens in the same polypeptide and tested using an enzyme-linked immunosorbent assay (ELISA) to detect different types of Chagas disease. rTC was able to discriminate between indeterminate (IND) and cardiac (CARD) cases and cross-reactive diseases, as well as healthy samples, with 98.28% sensitivity and 96.67% specificity, respectively. These data suggest that rTC has the potential to be tested in future studies against a larger serological panel for the diagnosis of Chagas disease.

## 1. Introduction

Chagas disease (CD) is a neglected tropical disease caused by the hemoflagellate *Trypanosoma cruzi* (*T. cruzi*) [1,2]. The infection occurs mainly via the vectorial route and is transmitted to humans via hematophagous insects of the subfamily *Triatominae* [3]. However, the parasite can also be transmitted congenitally, by blood transfusions, organ transplantation, or through the ingestion of contaminated food and beverages [3]. It is considered a public health problem, and there has been an increase in its global spread in recent years. Currently, the disease is endemic in 21 regions, and it is occurring in nonendemic countries, such as the United States of America, Canada, Spain, France, Switzerland, Italy, and Japan, as well as on other continents, such as Asia and Oceania [1,4].

According to the Pan American Health Organization (PAHO), about six to eight million people are infected with *T. cruzi* in the Americas, while about 70% of these people are living with the disease without knowing they are infected due to the absence of an appropriate diagnosis [5]. More than 10,000 people die every year from clinical complications of Chagas disease, and 75 million people are at risk of infection [4,6]. In Brazil, new cases were reported in most states, with 95% of them being concentrated in the northern region and 83% being reported in Pará state [7].

*T. cruzi* infection in humans results in an acute illness lasting six to eight weeks, which, if untreated, may evolve into a chronic phase [8,9,10]. In the acute phase, a local inflammatory lesion appears at the site of infection, wherein the parasite undergoes the first rounds of replication [11,12]. Afterward, the parasites spread through the body, with circulating trypomastigotes being easily found in the blood. During the chronic phase, circulating parasites cannot be detected through the inspection of blood; however, progressive tissue damage ensues, often with serious cardiac manifestations or gastrointestinal involvement. The chronic phase is characterized by a high production rate of specific immunoglobulin G (IgG) antibodies, and diagnosis at this stage relies mainly on serological techniques [13,14].

Parasitological and molecular diagnostic tests are the most appropriate methods during the acute phase. However, direct parasitological tests are not recommended during the chronic phase due to the absence of parasitemia [9,15,16]. The results of direct parasitological tests performed on biological fluids such as thick, fresh blood and smears are based on an analysis of the parasite in the blood of infected patients. However, these tests have low sensitivity and can generate false-negative results [17]. In addition, indirect parasitological tests, such as xenodiagnoses or blood culture, have low sensitivity varying between 20% and 50% and require assistance from other methods, such as the concentration test, to confirm the result. The enzyme-linked immunosorbent assay (ELISA) and Polymerase Chain Reaction (PCR) tests are indicated for use during the chronic phase because they are more sensitive and specific than parasitological tests [18]. Despite its high sensitivity and specificity, PCR is an expensive test and is difficult to standardize [19].

Immunoglobulin G antibody detection is the method of choice for diagnosis during the chronic phase of the disease because parasitemia is scarce. However, two different serological methods are recommended for diagnosis confirmation. Usually, the ELISA, indirect immunofluorescence, or indirect hemagglutination are employed [20]. ELISA techniques are generally based on crude extracts or antigens purified of epimastigote or trypomastigote forms. In addition, recombinant antigens, either alone or combined, and synthetic peptides are also widely used [21,22]. This assay is a widely used method for screening donors at blood banks in Brazil [23]. However, a gold standard for serological diagnosis has not yet been established. According to the PAHO, at least two different serological tests or two different *T. cruzi* antigens are required as serological discrepancies are common [24,25,26].

The chronic phases of CD can be divided into four clinical forms: (i) indeterminate form, (ii) cardiac form, (iii) digestive form, and (iv) mixed form when cardiac and digestive manifestations coexist simultaneously [27]. Individuals in the indeterminate form have a significant risk of developing chronic cardiomyopathy [28], and about 40% of individuals with chronic diseases may develop heart damage, digestive diseases, or both manifestations [29]. Therefore, the development of new markers capable of recording the recognition of different clinical forms and assessing the potential for progression and monitoring of the most severe form may contribute to the early adoption of therapeutic therapies [30,31].

The use of assays that employ a combination of recombinant antigens and the incorporation of different detection systems increase the sensitivity and specificity of the serological techniques [32]. The major advantage of recombinant antigen-based tests is that they minimize the extent of specificity problems [13,33]. We designed a recombinant multiepitope protein for diagnosing Chagas disease (rTC) and evaluated the discrimination of clinical forms and cross-reactivity. Our findings demonstrated that rTC can diagnose *T. cruzi*-infected individuals with low cross-reactivity and could be a valuable tool for producing immunodiagnostic kits for Chagas disease.

## 2. Materials and Methods

### 2.1. Strains, Reagents, and Samples

*Escherichia coli* (*E. coli*) BL21 (λDE3) and expression vector pET21a were purchased from Novagen (Merck, Darmstadt, Germany). HIS-Select^®^ Nickel Affinity Gel resin was used to purify rTC. Antibody-enzyme conjugates [monoclonal anti-poly histidine-horseradish Peroxidase (HRP) antibody and monoclonal anti-human IgG-HRP] were purchased from Sigma Aldrich (San Luis, MO, USA). Other reagents of analytical grade were obtained from standard commercial sources.

### 2.2. Serum Samples

The study was approved by the Human Research Ethics Committee of UFMG, with protocol numbers: CAAE–32343114.9.0000.5149/CAAE-48354315.8.0000.5149. CD-positive serum samples (n = 58) were classified according to the clinical form of the disease as described by Rocha et al. (2003) [34]: indeterminate form (IND, n = 30) for asymptomatic individuals who have sinus rhythm, without significant changes in the electrocardiogram, chest radiograph, and echocardiogram; and cardiac form 5 (CARD, n = 28) for individuals with echocardiographic signs of dilated left ventricle with systolic function impaired ventricular function, with or without manifestations of heart failure. Healthy individuals (n = 30) from a nonendemic area for the CD with negative characterization tests and individuals infected with leishmaniasis, visceral (n = 30), and cutaneous (n = 30), for cross-reactivity assessment, were included as the control group (NI). All sera were collected and characterized using two or more tests (indirect immune fluorescence, ELISA, or indirect hemagglutination).

### 2.3. Design of the Synthetic Gene, Cloning, and Expression

The synthetic gene was custom synthesized by GenOne (Rio de Janeiro, Brazil) with codon usage for *Escherichia coli* and cloned as an *NdeI/XhoI* fragment into pET21a in-frame with a C-terminal histidine tag to allow protein purification via affinity chromatography. The resulting plasmid was used to transform *E. coli* BL21 (DE3) *plysS* competent cells and selection was performed on LB agar plates containing 100 µg/mL ampicillin. The DNA and amino acid sequences for the entire synthetic gene construct are proprietary (under Brazilian patent No. BR1020220183139) and are not available to be shared at this stage. An individual colony was inoculated in 5 mL LB (10 g/L Casein Peptone, 5 g/L Yeast extract, 10 g/L NaCl, pH 7.2) containing 100 g/mL ampicillin and allowed to grow overnight at 37 °C under agitation (200 rpm). At one point, twenty-five mL of the pre-culture was transferred to 25 mL LB in a 250 mL Erlenmeyer flask. The culture was grown in these conditions until an OD600 of 0.6, at which point 1 mM IPTG was added. Aliquots were withdrawn at 0.5, 1.5, and 2.5 h after induction. The induced culture was harvested using centrifugation at 6000× *g* for 15 min at 4 °C and the pellet was stored at −80 °C.

### 2.4. Purification of the rTC

Our purification strategy involved adding lysis buffer (8M urea, 50 mM NaH_2_PO_4_, 300 mM NaCl, and 10 mM imidazole, at pH 8.0) to the cellular pellet and lysing cells with lysis buffer overnight at 4 °C. The lysed cells were then sonicated, and 500 μL of Ni-NTA resin (Sigma Aldrich) was then added to the supernatant for batch purification. The resign was washed with urea-containing buffer (8 M urea, 50 mM NaH_2_PO_4_, 300 mM NaCl, 5 mM imidazole, and pH 8.0), and rTC proteins were eluted with imidazole-containing buffer (with 8 M urea, 50 mM NaH_2_PO_4_, 300 mM NaCl, and 100 mM imidazole at pH 8.0).

### 2.5. Electrophoresis and Western Blotting

Protein integrity and molecular mass calculation were evaluated using running samples on 12% SDS-PAGE. Proteins were stained with Coomassie Brilliant Blue R-250 (Sigma Aldrich). Following electrophoresis, the proteins were transferred electrophoretically to a Polyvinylidene difluoride (PVDF) membrane for Western blotting. The membrane was blocked with 5% skim milk powder in phosphate-buffered saline (PBS) for 2 h at room temperature. The blot was then washed three times with PBS containing 0.1% Tween 20 (PBS-T) and incubated with monoclonal mouse anti-His HRP (horseradish peroxidase, Sigma Aldrich), diluted 1:1000 in PBS for 2 h at room temperature. Following three washes with PBST, the specific protein band was visualized using the 3,3′-Diaminobenzidine tetrahydrochloride detection method.

### 2.6. ELISA Assay

The immune enzymatic assay was used to detect specific binding of the recombinant protein rTC to anti-CD antibodies. The wells of the polystyrene plates (Costar, NY, USA) were sensitized with 8.75 ng of purified rTC dissolved in 100 µL of 0.1 M sodium carbonate-bicarbonate buffer (pH 9.6). After incubation at 4 °C for 16 h, the coated wells were washed with PBST and blocked for 1 h at 37 °C with PBS buffer (pH 7.2) containing 5% (*w*/*v*) dried skim milk powder and washed again with PBS-T. Subsequently, 100 µL of a dilution (100 µL PBST, 5% [*w*/*v*] dried skim milk powder, and 1 µL serum) was placed into the wells, resulting in a final dilution of approximately 1:100. After incubation for 30 min at 37 °C, the wells were washed with PBST and 100 µL of goat anti-human immunoglobulin G conjugate peroxidase-labeled (Sigma Aldrich) and diluted at 1:40,000 in PBS buffer (pH 7.2), which was added following incubation for 30 min at 37 °C. The wells were again washed, and 100 µL 3,3,5,5-tetramethyl-benzidine substrate (BD Biosciences, Franklin Lakes, Nova Jersey, USA) was added and incubated for 15 min at room temperature in the dark. Reactions were stopped by the addition of 100 µL of sulfuric acid (0.2 M). The optical densities were read at 450 nm.

### 2.7. Statistical Analyses

The cut-off values of the in-house ELISA assay were established for optimal sensitivity, specificity, and accuracy (area under the curve) using receiver operating characteristic (ROC) curve analysis in GraphPad Prism (Boston, MA, USA). The absorbance readings produced by the ELISA assay were compared using the Mann–Whitney U test and the Kruskal–Wallis test, considering a level of significance of *p* < 0.05.

## 3. Results

### 3.1. Design, Expression, and Purification of the rTC

**Figure 1:**
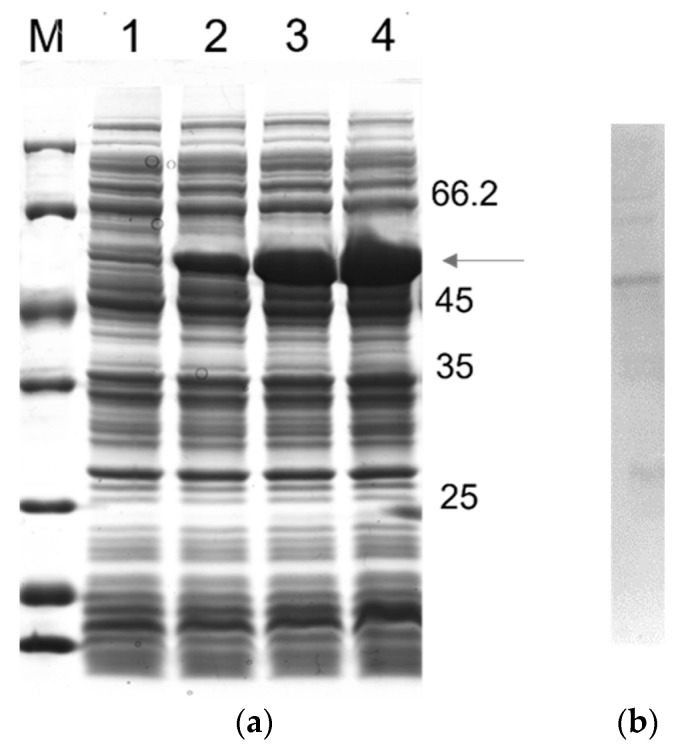
Time course rTC expression in *E. coli* BL21 (λDE3) and analysis using SDS-PAGE 12%: (**a**) Lane M, unstained protein molecular mass marker (Thermo Scientific); Lane 1, noninduced control; Lanes 2–4, after 0.5, 1.5, and 2.5 h of induction, respectively. (**b**) Western blotting analysis. The monoclonal antibody, anti-polyhistidine conjugated horseradish peroxidase (HRP), was diluted 1:1000 in PBS.

**Figure 2:**
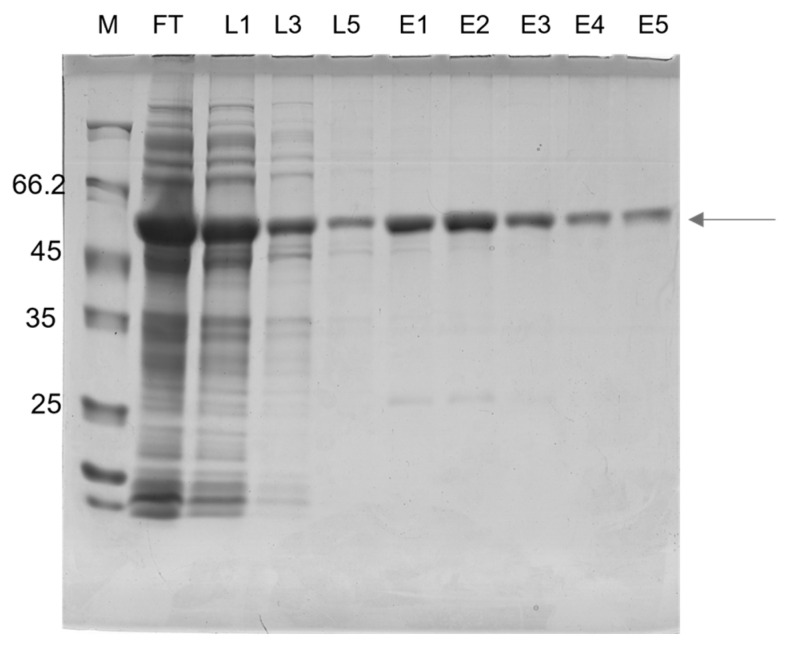
Analysis of the rTC using 12% SDS-PAGE after the purification procedure. Fractions were collected after Ni-NTA chromatography. Lane 1: M, molecular mass markers: Unstained Protein Weight Marker Standards (Thermo Scientific). Lane 2: FT: flow-through (unbound fraction); Lanes 3–5: L1, 2 e 3: wash steps; Lanes 6–10: E1, 2, 3, 4 e 5: purified rTC after elution with 100 mM imidazole.

To design a multiepitope protein that could be of diagnostic use, conserved epitopes, which are known to elicit anti-CD antibodies, were selected based on data from the literature. The antigenic epitopes chosen were assembled and connected using flexible glycine-serine linkers (GSGSG). This allowed the epitopes to be freely available for interaction with their cognate antibodies, thus contributing to the overall sensitivity and specificity of the diagnostic test. Our chosen resultant protein (rTC) is a construct in which the selected epitopes join in tandem, and it has an apparent size of ~50 kD; moreover, a 12% SDS-PAGE gel showing a time course of rTC expression in *E. coli* after IPTG-induced protein induction, which was stained via Coomassie Blue, is shown in Figure 1a. The main ~50 kD band (Figure 1a) was shown via Western blot to have a polyhistidine tag (Figure 1b). We further confirmed that we could purify rTC via affinity (Ni-NTA) chromatography (Figure 2).

### 3.2. ELISA—Human Anti-IgG Chagas Recognized rTC

**Figure 3:**
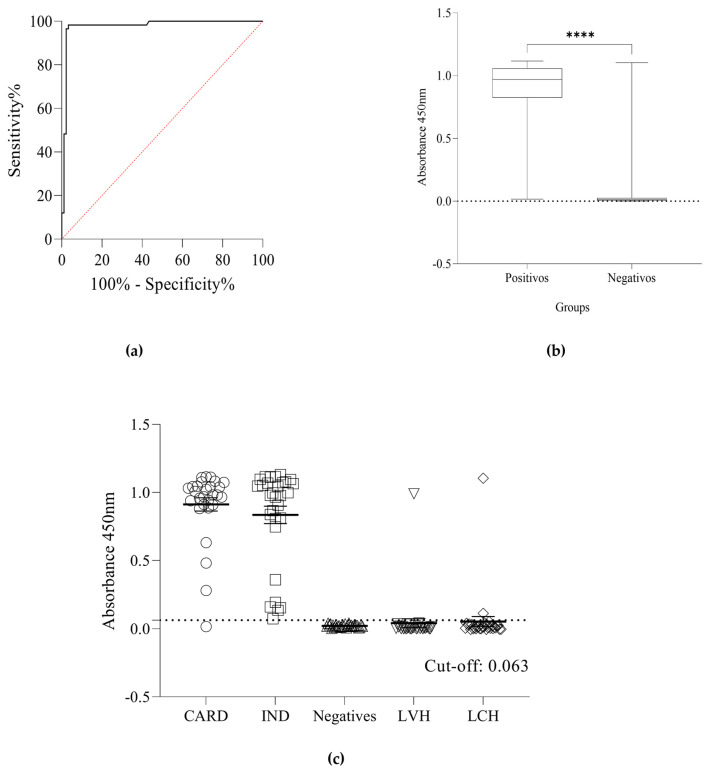
(**a**) ROC curve showing the in-house ELISA’s area under the curve (AUC). (**b**) Boxplots of the absorbance readings from the sera in the positive and negative groups presented statistically significant differences, the asterisk indicates statistical significance (**** *Mann-Whitney U*: *p* < 0.0001). The median of absorbance readings obtained using the in-house ELISA in the positive group was 0.969, 25% percentile = 0.825, 75% percentile = 1.059. In contrast, the median of absorbance readings obtained using the in-house ELISA in the negative group was 0.012, 25% percentile = 0.004, 75% percentile = 0.024. (**c**) Reactivity and positivity of the in-house ELISA were measured in serum samples of positive groups (cardiac form (CARD) n = 28 and indeterminate form (IND) n = 30) and negative controls groups (NI), healthy individuals (HI) n = 30, human visceral leishmaniasis (LVH) n = 30, and human cutaneous leishmaniasis LCH n = 30). The cut-off was determined as 0.063. The sensitivity and specificity were 98.28 (95% CI: 90.86–99.91) and 96.67% (95% CI: 90.65–99.09%) respectively. Three samples from the negatives group showed false-positive results. In the Kruskal–Wallis test, we did not observe a significant difference between the diagnosis (*p* > 0.9999) of the IND and CARD clinical forms, only with NI (*p* < 0.0001).

**Figure 4:**
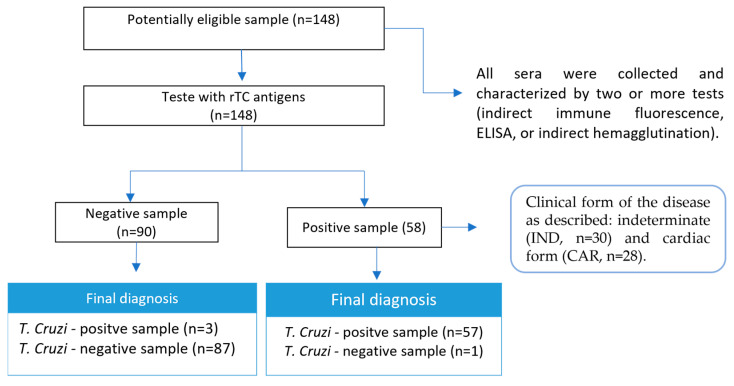
Study design. Only a sample of the group of patients with the cardiac form did not react to the assay using rTC as an antigen. Clinical forms of the disease classified according to Rocha et al. [35].

Since the underlying goal of this study was the development of a recombinant multiepitope protein that could be used in a Chagas diagnosis kit, the ability of rTC to detect anti-CD antibodies was tested using an in-house ELISA. The cut-off value of 0.063 was defined using an analysis of the receiver operating characteristic (ROC curve), and the area under the curves was 0.97 (95% CI 0.93 to 0.99) (Figure 3a). The median of the absorbance readings produced using the in-house ELISA presented a statistically significant difference (*p* < 0.0001) between the positive and negative groups (Figure 3b). rTC was able to detect both indeterminate and cardiac forms with a minimal cross-reaction for human visceral leishmaniasis (LVH) and human cutaneous leishmaniasis (LCH) (Figure 3c). The sensitivity and specificity values were 98.28% (57/58) and 96.67% (87/90) (Figure 4), respectively, and the positive likelihood ratio from the assay was 29.48. These results show the ability of rTC to distinguish positive and negative human sera for IgG and its potential for use in diagnosis kits.

## 4. Discussion

The diagnosis of CD is essential to ensure that patients will receive adequate chemotherapy. This paper describes the development of a recombinant multiepitope protein and shows its performance in the serological diagnosis of Chagas disease. rTC can detect individuals infected with Chagas disease in both IND and CARD forms, presenting a sensitivity of 98.28%, a specificity of 96.67%, and an area over the curve (AUC) of 0.977.

Currently, there is no gold standard for the diagnosis of CD, requiring at least two distinct serological assays. Different ELISAs for detecting Chagas are commercially available because there is no universal antigen for the diagnosis, and variations of performance when testing samples from different countries have been reported [35,36].

Different factors may be associated with the performance of the test, such as the intrinsic characteristic of each antigen and the use of the assays in countries and regions where the genetic strains differ from those used for the development of the antigen [37,38,39]. To overcome this problem, a multiepitope-based protein could be used as an alternative to crude antigens, improving sensitivity and specificity and reducing production costs [40,41]. The construction of such recombinant proteins containing high-density epitopes has been a preferred alternative for the diagnosis of diseases given that these proteins exhibit a broad ability to expose their epitopes more efficiently, resulting in improved sensitivity and specificity [40,41].

In this work, a multiepitope protein was developed and evaluated as a strategy for the diagnosis of CD, focusing on antigens and conserved proteins to construct the encoding rTC. Such antigens were evaluated using information available in the literature, with the main B-cell epitopes being selected based on the following criteria: (i) immunodominance, (ii) specificity for anti-CD antibodies, (iii) straight chain, and (iv) phylogenetic conservation in most genotypes in circulation in different parts of the world [40,41,42,43].

Several multiepitope proteins for the diagnosis of different diseases, both human [40,41,42,44,45,46,47] and animal [48,49], have been described and show promising results in terms of increasing and improving sensitivity and specificity and reducing cross-reactivity, indicating that multiepitope technology is efficient for the detection of different pathologies. Few studies in the literature have used recombinant multiepitope to diagnose CD. A search conducted at Pubmed using the keywords “Multiepitope protein” and “diagnosis” identified 270 published articles. Out of these, only four were related to the diagnosis of CD and presented sensitivity and specificity data [38,50,51,52,53]. However, none of them evaluated the potential of their antigens to detect both chagasic forms classified with the clinical form, as performed in this study.

Houghton et al. (2000) [50] described the use of a single multiepitope peptide, 2/D/E, formed by sequences of antigens TcD, TcE, and PEP-2. This protein was highly sensitive, detecting 239 out of 240 consensus-positive sera, indicating a sensitivity of 99.6%. In contrast, of the 149 test serum samples obtained from healthy, random donors, 148 were below the designated cutoff, indicating a specificity of 99.33%. Camussone et al. (2009) [51] reported the use of two multiepitope-based proteins, CP1 and CP2, tested separately as antigens to detect CD. The discrimination efficiency values obtained for CP1 and CP2 were 25% and 52% higher than those of their antigens. CP2 was the only multiepitope protein that showed discrimination efficiency between negative and positive samples of CD, showing 98.6% sensitivity and 99.4% specificity. Duthie et al. (2013) [38] studied the use of multiepitope fusion proteins TcF, TcF43, and TcF26 for the diagnosis of CD. The authors demonstrated that the fusion proteins TcF26 and TcF43 yielded strong responses against sera that were also strongly detected by TcF, one of the recombinant antigens currently used with tests for CD. Conversely, Peverengo et al. (2018) [52] tested the use of multiepitope proteins CP1 and CP3 to diagnose chronic CD. The sensitivity of CP3 and CP1 was 100% and 90.2%, respectively, and specificity was 92.5% and 100%, respectively, showing that both multiepitope proteins detect chronic *T*. *cruzi* infection.

Despite not having an established treatment for chronic infections, the evaluation of the treatment results depends on serological monitoring [54]. Therefore, the development of new biomarkers is essential to identify the disease in its acute or chronic phase [34]. Nonparametric analysis was performed for more than two groups to determine whether rTC was able to distinguish between clinical forms; however, no significant difference was observed between the diagnosis (*p* > 0.9999) of the IND and CARD clinical forms. However, the diagnosis of IND individuals is relevant for the management and care of CD. Intervention in the initial phase of Chagas disease (acute and undetermined phase) can favor a reduction in transmission, improve the health condition of infected individuals, and save money on the cost of treatment [34,40].

The results of the present study differ from the one conducted by Longhi et al. (2012) [31], in which a recombinant J7 protein and several peptides were evaluated for their diagnostic capacity, considering clinical, electrocardiographic, and radiological results. They were then classified as indeterminate (IC), digestive (CD), mild cardiomyopathy (MCC), and severe cardiomyopathy (SCC) forms of CD. The authors found that the anti-JL7 Ab concentration (using optical density [OD]) was higher in sera from patients with SCC as compared to those with indeterminate form (IC) (*p* < 0.019), suggesting that this antigen could be used as a prognostic marker for the indeterminate form (HF).

Another important factor for an accurate diagnosis is the absence of cross-reactivity, especially between CD and leishmaniasis. Our study results showed low cross-reactivity with visceral (1/30) and tegumentary (2/30) leishmania. A similar result has been described by Daltro et al. (2019) [55], in which four chimeric antigens were evaluated to assess cross-reactivity with American tegumentary or visceral leishmaniasis. The chimeric antigens exhibited poor seropositivity for leishmaniasis infection compared to the evaluated commercial immunoassays.

## 5. Conclusions

In conclusion, rTC showed satisfactory performance in the diagnosis of CD, both in its chronic phase and for individuals with the IND form; moreover, it proved to be effective for possible application in Chagas diagnostic kits. The development of more accurate tests that consider the genetic variability of *T. cruzi* and present low cross-reactivity may reduce the costs of diagnosis. Nevertheless, further studies using a greater serological panel, and including samples from other countries or regions, are essential to effectively evaluate the maintenance of the diagnostic performance of rTC and the capacity of a diagnosis in acute-phase samples from treated patients for assessing rTC as a prognostic marker.

## 6. Patents

One patent resulting from the work reported in this manuscript is under protection at Instituto Nacional de Propriedade Industial (INPI): Brazilian patent No. BR1020220183139.

## Data Availability

Not applicable.
